# Atractyloside Protect Mice Against Liver Steatosis by Activation of Autophagy *via* ANT-AMPK-mTORC1 Signaling Pathway

**DOI:** 10.3389/fphar.2021.736655

**Published:** 2021-09-21

**Authors:** Pengfei Zhang, Xinyu Cheng, Huimin Sun, Yajing Li, Wuxuan Mei, Changchun Zeng

**Affiliations:** ^1^ Department of Medical Laboratory, Shenzhen Longhua District Central Hospital, The Affiliated Central Hospital of Shenzhen Longhua District, Guangdong Medical University, Shenzhen, China; ^2^ Clinical Medical College, Hubei University of Science and Technology, Xianning, China

**Keywords:** atractyloside, adenine nucleotide translocase, autophagy, lipid degradation, nonalcoholic fatty liver disease

## Abstract

**Objective**: Adenine nucleotide translocase (ANT) can transport ADP from cytoplasm to mitochondrial matrix and provide raw materials for ATP synthesis by oxidative phosphorylation. Dysfunction of ANT leads to limitation of ADP transport and decrease of ATP production. Atractyloside (ATR) is considered as a cytotoxic competitive inhibitor binding to ANT, making ANT vulnerable to transport ADP, and reduces ATP synthesis. Moreover, the blockage of ANT by ATR may increase ADP/ATP ratio, activate AMPK-mTORC1-autophagy signaling pathway, and promote lipid degradation in steatosis hepatocytes. The present study was conducted to investigate the mechanism of ATR, regulate ANT-AMPK-mTORC1 signaling pathway to activate autophagy, and promote the degradation of lipid droplets in high-fat diet (HFD) induced liver steatosis.

**Methods**: ICR mice were fed with HFD for 8 weeks to induce liver steatosis, and ATR solution was given by intraperitoneal injection. Intracellular triglyceride level and oil red O staining-lipid droplets (LDs) were assessed, the expression of proteins related to ANT-AMPK-mTORC1 signaling pathway and autophagy were determined, and the colocalization of LC3B and Perilipin 2 was performed.

**Results**: ATR treatment decreased the serum AST level, relative weight of liver and epididymal fat, and body weight of HFD mice. The LDs in HFD mice livers were reduced in the presence of ATR, and the TG level in serum and liver of HFD mice was significantly reduced by ATR. In addition, ATR inhibited ANT2 expression, promoted the activation of AMPK, then increased Raptor expression, and finally decreased the mTOR activity. Furthermore, ATR increased the protein level of LC3A/B and ATG7, and a strong colocalization of LC3B and PLIN2 was observed.

**Conclusion**: ATR treatment blocks ANT2 expression, promotes the activation of AMPK, then decreases the mTOR activity, and finally promotes autophagosomes formation, thus accelerating the degradation of HFD-induced accumulated lipids in the liver. This will provide new therapeutic ideas and experimental data for clinical prevention and treatment of non-alcoholic fatty liver disease.

## Introduction

Nonalcoholic fatty liver diseases (NAFLD), a liver disease characterized by steatosis of hepatic parenchymal cells and the accumulation of excessive lipid in the liver, is generally accompanied by hyperglycemia, hypertension, fatty liver, and other diseases (Persson and Bondke Persson, 2018). The clinical treatments for NAFLD include improving insulin resistance, which prevents metabolic syndrome, and reducing liver lipid deposition, which leads to steatohepatitis and decompensation of liver function. Diet control and physical activity are the first-line treatment in NAFLD, but most of them achieve limited success, especially in the disorder of liver lipid metabolism caused by genetic susceptibility. In short, exploring a treatment that can directly degrade the accumulated lipids in hepatocytes is the key treatment for NAFLD (Madrigal-Matute and Cuervo, 2016; Gluchowski et al., 2017; Neuschwander-Tetri, 2020).

Adenine nucleotide translocase (ANT) is a solute carrier that specifically exchanges ADP and ATP through the mitochondrial inner membrane by antiport mechanism (Ruprecht et al., 2019). Thus, the function of ANT is essential for coupled respiration (and/or uncoupled respiration) leading to ATP production in mitochondria (Stephane et al., 2019). Recently, ANT-induced uncoupling respiration was found to be markedly increased in ob/ob mice and high-fat diet (HFD) induced obesity mice. However, knockout of ANT systematically or liver specifically significantly decreased the body weight and hepatic steatosis, increased the insulin sensitivity, and reduced the inflammation in obese mice (Seo et al., 2019). Thus, specific knockout or pharmacological inhibition of ANT may have a potential for NAFLD treatment.

Atractyloside (ATR), a diterpenoid glycoside with one isovaleric and two sulfate groups, is a high-affinity specific inhibitor of the mitochondrial ADP/ATP carrier (Kedrov et al., 2010). ATR competitively binds to ANT, inhibits the transport of ADP across the mitochondrial membrane, prevents the synthesis of ATP, and leads to a failure in energy balance, which is considered to be cytotoxic when ATR is at a high concentration. However, ATR has a weak inhibitory effect on ANT at low concentration, which only affects ATP synthesis to a certain extent and can improve the ADP/ATP ratio of cells (Stewart and Steenkamp, 2000). In theory, altered cellular energy expenditure may activate adenosylate-activated protein kinase (AMPK, an energy receptor in cells) and in turn inhibit mammalian rapamycin (mTOR) activity and activate autophagy, which may promote the degradation of stored lipids in lipid droplets and may be useful in the treatment of NAFLD (Stephane et al., 2019). Additionally, the analog of ATR, Carboxy-ATR, can also improve the degree of obesity and hepatic steatosis in mice (Cho et al., 2017) by inhibiting ANT in a noncompetitive manner (Stewart and Steenkamp, 2000). This evidence suggested that ATR is a safer compound and has a potential treatment effect on NAFLD. Moreover, we have previously reported that low concentrations (2.5, 5, and 7.5 μM) of ATR treatment could activate autophagy to accelerate the degradation of TGs in steatosis HepG2 cells; the mechanism may be related to the activation of the AMPK/mTOR pathway induced by the increased ADP/ATP ratio (Zhang et al., 2020). Therefore, it is speculated that ATR also could exert its effect to improve hepatic steatosis of mice feed with a high-fat diet.

To prove this hypothesis, the present study investigated the effect of ATR on ANT-AMPK-mTORC1 signaling pathway, autophagy, and steatosis level in the liver of HFD fed mice. The results showed that ATR treatment blocked ANT2 expression, promoted the activation of AMPK, then decreased the mTORC1 activity, finally promoted autophagy activation, and thus accelerated the degradation of HFD-induced accumulated lipids in the liver. This will provide new therapeutic ideas and experimental data for clinical prevention and treatment of NAFLD.

## Methods

### Atractyloside Solution Preparation

ATR (SA9830, 97% pure) was purchased from Solarbio Life Science (Beijing, China). ATR was dissolved in dimethyl sulfoxide (DMSO) at a concentration of 2 mg/mL as a stock solution; then, the working solution was diluted at concentrations of 0.1 mg/ml and 0.2 mg/ml, respectively. The dose of ATR (2 and 4 mg/kg of body weight) used in this study was chosen according to the previous study (Zhang et al., 2020).

### Animals Handling and Atractyloside Treatment

All experimental procedures performed in this study were approved by the Animal Care and Use Committee (Approved number: GDY2002225, date: August 19, 2020) of Guangdong Medical University (Zhanjiang, China).

32 male ICR wide-type (WT) mice (6–8-week-old, 40–45 g) were obtained from Experimental Animal Center of Guangdong Medical University (Guangdong, China, production license: SCXK (Yue) 2018-0008) and allowed to acclimatize to the animal facility environment for a week before the experiment. Four mice were housed per cage in a specific pathogen-free laboratory (temperature 24 ± 2°C, humidity 50 ± 5%, and a standard 12 h light/dark cycle) with access to food and water. A total of 32 mice were distributed according to a single factorial arrangement. All mice were randomly divided into 4 treatment groups (8 mice per group and 4 mice per cage), including the normal control group, HFD control group, HFD with low ATR group (2 mg/kg ATR), and HFD with high ATR group (4 mg/kg ATR). Control mice were fed with a maize- and soybean-based basal diet (16.7% calories from fat, 3.77 Kcal/g diet), and mice in the other three groups were feed with a high-fat diet (GD60 rodent diet, Table 1, 60% calories from fat, 5.24 Kcal/g diet). All the food was purchased from Guangdong Animal Laboratory Animal center. The total feeding duration was 56 days (8 weeks), and ATR solution was given by intraperitoneal injection at a volume of 0.4 ml for 2 weeks before the feeding duration ends. Feed and water were provided *ad libitum* throughout the experiment.

**TABLE 1:** Composition and nutrient levels of high-fat diet (GD60 Rodent Diet).

Ingredient (g/kg as fed)	Mass	Energy (Kcal)
Casein, 30 mesh	200	800
L-Cystine	3	12
Corn starch	0	0
Maltodextrin 10	125	500
Sucrose	63.8	275.2
Cellulose, BW200	50	0
Soybean oil	25	255.2
Lard	245	2205
Mineral mix S10026	50	36
Vitamin mix AIN-76A	10	39
Choline bitartrate	2	0
Total	773.8	4,072.2
Typical analysis	Mass (%)	Energy (%)
Protein	26	20
Carbohydrate	26	20
Fat	35	60
Total		100

### Record of Body Weight, Food Intake, Relative Weight of Liver and Epididymal Fat

The body weights of mice were recorded every 3 days from the 7th week to the 8th week, and the food consumption of each cage was recorded weekly. The final liver weight and epididymal fat weight were also recorded and the results were expressed as a relative weight to body weight (g/kg of body weight).

### Serum Biochemical Indices Analysis

At the end of the experiment, about 1 ml blood samples were collected from the angular vein of mice under anesthesia by pentobarbital. Serum samples were obtained by centrifugation at 1790 × g for 10 min at room temperature and later stored in microtubes at −80°C. The levels of alanine aminotransferase (ALT), aspartate aminotransferase (AST), triglyceride (TGs) were estimated using commercially available kits purchased from Nanjing Jiancheng Bioengineering Institute (Nanjing, Jiangsu, China) in accordance with the manufacturer’s instruction.

### Hepatic Lipid Determination and Histological Analysis

Each mouse’s liver was collected and placed in a cryogenic vial after being washed with ice-cold sterile saline solution to remove blood contamination. For the determination of hepatic lipid contents, about 0.5 g of liver was used to prepare the homogenate. The minced tissue was homogenized in an ice-cold phosphate buffer solution (w/v, 1:9) using a glass homogenate tube and was centrifuged at 1,346 × g for 10 min at room temperature, and then the supernatant was collected and stored at −80°C until further analysis. The TG level in each sample was estimated using a commercially available kit purchased from Nanjing Jiancheng Bioengineering Institute (Nanjing, Jiangsu, China) in accordance with the manufacturer’s instructions. The protein concentrations of the tissue homogenates were determined *via* bicinchoninic acid assay (Walker, 2009).

For the histological analysis, liver tissues were fixed with 4% paraformaldehyde in PBS for 10 min at room temperature. The fixed liver samples were embedded with optimal cutting temperature compound (OCT); then, each sample was used to prepare 5 slides and each slide had 3 sections (10 μm thickness), which were stained with hematoxylin and eosin (H&E) and then sealed by a neutral resin size. For oil red O staining, the liver slides were stained with freshly prepared oil red O solution for 10 min. Afterward, the slides were washed thrice with distilled water and imaged under an inverted light microscope (Axio Vert. A1, Carl Zeiss, Germany). The ratio of oil red O was subjected to densitometric analysis based on the average percent of LDs accumulated hepatocytes per field at 200 magnification in 20 random fields by ImageJ 1.45 software (NIH, United States). For the NAFLD activity score (NAS), the liver slides were each read twice by 3 pathologists from the pathology department of our hospital. Weighted kappa scores were used to measure the degree of inter-rater and intra-rater agreement between and within multiple pathologists. The NAS is defined as the unweighted sum of the score of steatosis (0–3), lobular inflammation (0–3), and ballooning (0–2). Thus, the score is ranging from 0 to 8 (Kleiner et al., 2005). In brief, the severity of steatosis was graded from 0 to 3 (0 for <5%, 1 for 5–33%, 2 for 33–66%, 3 for >66%). Ballooning was determined by the number of prominent ballooning cells and scored from 0 to 3 (0 for none, 1 for few balloon cells, 2 for many cells/prominent ballooning). The lobular inflammation was assessed by counting the number of inflammatory foci per 200× magnification field in 20 random fields and scored 0–3 (0 for no foci, 1 for <2 per field, 2 for 2–4 per field, and 3 for >4 per field. The fibrosis was scored 0–4 (0 for no fibrosis, 1 for perisinusoidal or periportal fibrosis, 2 for perisinusoidal and portal/periportal fibrosis, 3 for bridging fibrosis, and 4 for cirrhosis). Notably, fibrosis is less reversible and generally thought to be a result of disease activity, which is not included as a component of the activity score (Kleiner et al., 2005). Cases with NAS of 0–3 were largely considered not diagnostic with steatohepatitis; on the other hand, cases with a score of >4 were diagnosed as steatohepatitis, and the scores between 3 and 4 were diagnosed with the possibility of steatohepatitis. Additionally, cases without ballooning, lobular inflammation, and fibrosis, but with the ratio of steatosis more than 33%, were diagnosed as NAFLD, and cases with the ratio of steatosis between 5 and 33% were diagnosed as hepatocytes steatosis.

### Western Blotting Analysis

The total protein samples were prepared by extracting liver in RIPA lysis buffer (Beyotime Biotechnology, Jiangsu, China), followed by centrifugation at 12,000 rpm for 15 min at 4°C. Supernatants containing total proteins were quantified with a BCA protein estimation kit (Meilun Biotechnology, Dalian, China) according to the previous method (Walker, 2009), and 50 μg of the total protein samples was resolved with 8, 10, or 15% polyacrylamide gel (depending on the molecular size of the proteins to be analyzed). Thereafter, immunoblotting was performed by transferring the resolved protein onto a PVDF membrane in a trans-buffer at 100 V for 1 or 2 h depending on the molecular size of the protein. Furthermore, the PVDF membranes were blocked with 5% skimmed milk or 5% BSA (for phosphorylated protein) in TBST buffer (20 mM Tris-HCl, pH 7.5, 150 mM sodium chloride, and 0.05% Tween 20) for 2 h, washed thrice with TBST buffer for 5 min each, and incubated overnight with primary antibodies including ANT2 (A15639, ABclonal), AMPKα (5831, CST), phospho-AMPKα (2535, CST), Raptor (2280, CST), mLST8 (3274, CST), mTOR (2983, CST), phospho-mTOR (2974, CST), SQSTM1/p62 (23214, CST), LC3A/B (3868, CST), and ATG7 (8558, CST) at 4°C. Afterward, the membranes were washed thrice with TBST buffer for 5 min each time and incubated with anti-rabbit or anti-mouse (depending on the species of primary antibody) secondary antibodies conjugated to horseradish peroxidase (1:2,000) for 2 h at room temperature. The blots were developed in the dark by using an ECL detection kit. The developed blots were subjected to densitometric analysis using ImageJ 1.45 software (NIH, United States) and normalized with GAPDH (5174, CST) as the internal control.

### Colocalization of LC3B Protein With Perilipin 2 Protein

The liver slides were prepared as before; the fixed liver slides were permeabilized with 0.1% Triton X-100 for 10 min and then washed and blocked with 2% BSA for 1 h at room temperature. After blocking, the slides were incubated with LC3B (83506, CST) mouse antibody (1:300) overnight at 4°C, followed by incubation with the Cy3 goat anti-mouse IgG (AS008, ABclonal) secondary antibody (1:500) for 1 h at room temperature. Furthermore, the slides were incubated with perilipin 2 (PLIN2; A6276, ABclonal) rabbit antibody (1:200) overnight at 4°C, followed by incubation with the FITC goat anti-rabbit IgG (ab6717, Abcam) secondary antibody (1:500) for 1 h at room temperature. Finally, the slides were counterstained with DAPI for 5 min to stain the nucleus. The coverslips were mounted on slides and visualized under a confocal microscope equipped with 493, 550, and 455 nm lasers (LSM 800, Carl Zeiss, Germany).

### Statistical Analysis

Quantitative data were presented as the mean ± SD and statistically evaluated *via* one-way ANOVA followed by Tukey’s multiple comparison test using the Graph Pad Prism 6 software (GraphPad software, San Diego, CA, United States). In the data analysis, *p* < 0.05 was considered statistically significant.

## Results

### Body Weight, Relative Weight of Organs, and Serum ALT and AST Level Changes

**FIGURE 1:**
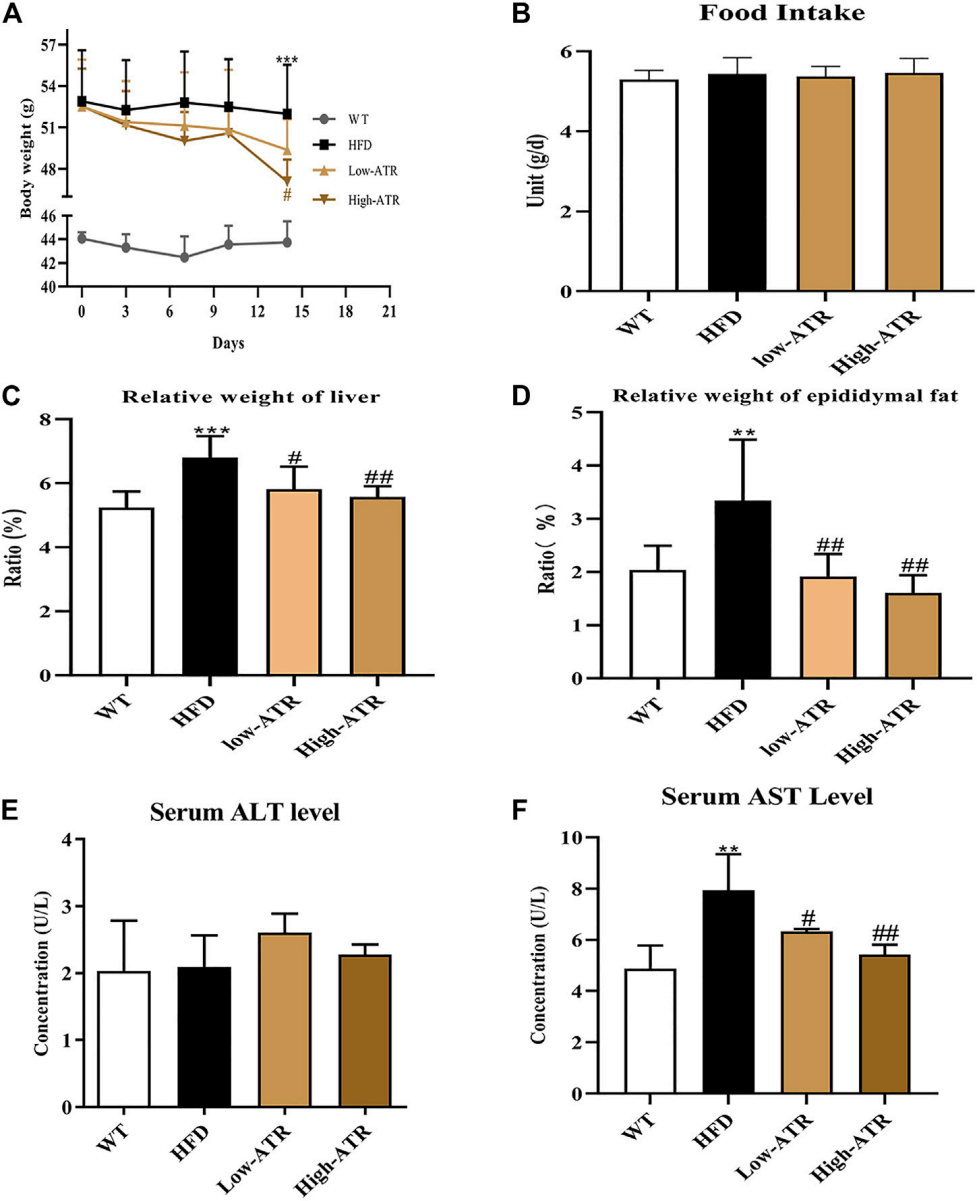
Changes of body weight, relative weight of organs, and serum ALT and AST level in HFD mice. **(A)** 14-day body weight change. **(B)** Food intake. **(C)** Relative weight of liver (g/kg). **(D)** Relative weight of epididymal fat (g/kg). **(E)** Serum ALT level. **(F)** Serum AST level. Data are expressed as mean ± SD (n = 8). *p < 0.05, **p < 0.01, ***p < 0.001 (* represents HFD groups compared with the WT group). ^**#**^ *p* < 0.05, ^**##**^ *p* < 0.01, ^**###**^ *p* < 0.001 (^**#**^ represents ATR treatments compared with the HFD group). WT = control group, and HFD = high-fat diet group.

The body weights of HFD mice were significantly higher than WT mice. Starting from the 4th day, injection of ATR compromised the increased body weight of HFD mice, and the body weight was significantly decreased by a high concentration of ATR at the end of the experiment (14th day; Figure 1A). In contrast to the WT group, HFD induced the relative weight (RW) of liver and epididymal fat, but ATR inclusion reversed the increased RW of those internal organs (*p* < 0.05; Figures 1C, D). The serum AST level was markedly increased in the HFD group, but the lower level of AST was observed in the ATR group as compared with the HFD group (*p* < 0.05; Figure 1F). No significant impact of ATR on food intake (Figure 1B) and serum ALT level was found (*p* > 0.05; Figure 1E). These findings suggested that ATR can reverse the changes of body weight, RW of liver and epididymal fat, and the serum AST level induced by HFD.

### Atractyloside Decreased the Lipid Content of High-Fat Diet Feed Mice

**FIGURE 2:**
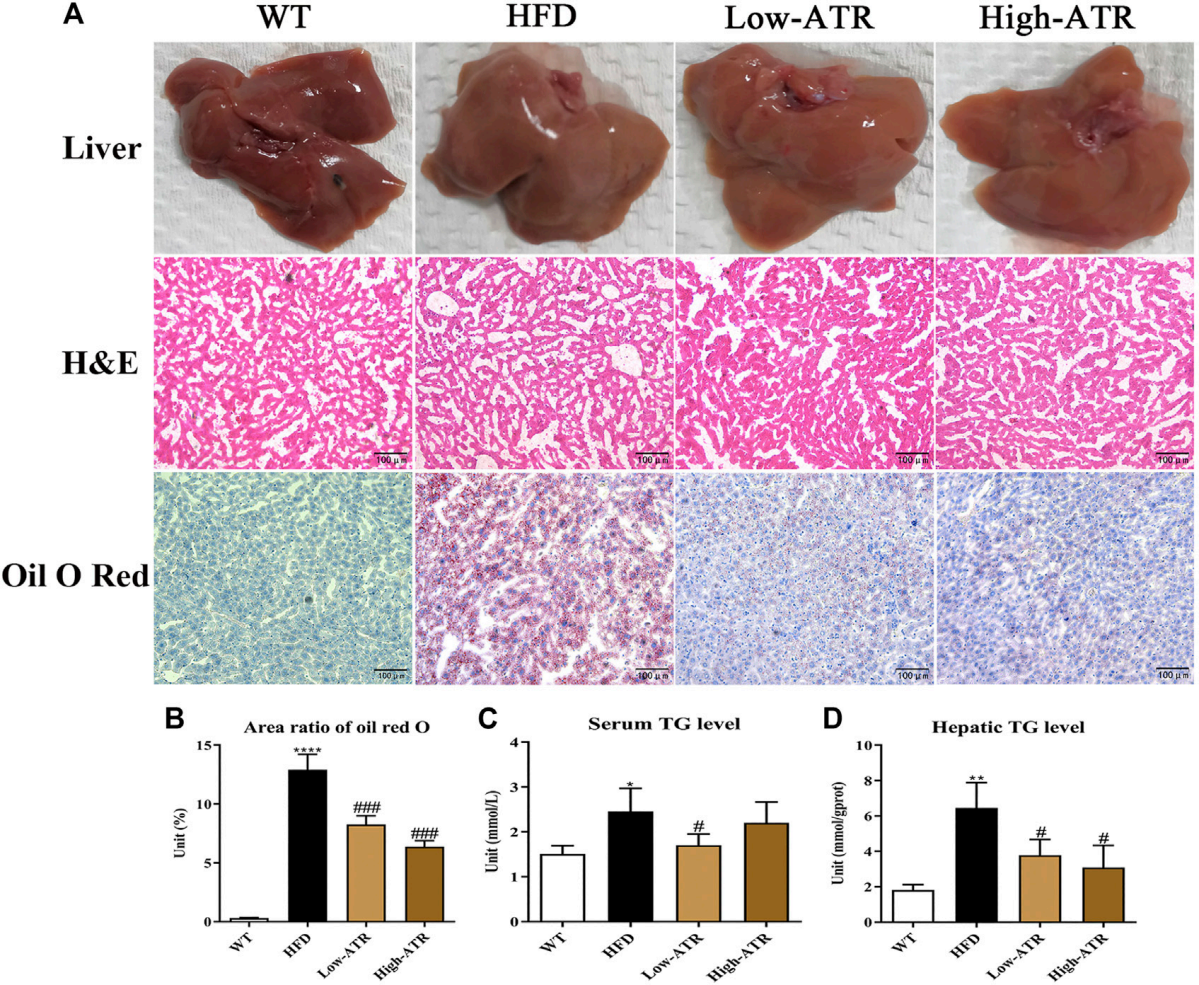
The changes of liver morphology and hepatic lipid content. **(A)** The fresh liver sample, H&E staining, and oil red O staining of liver. **(B)** Area ratio of oil red O in the liver. **(C)** Serum TG (triglycerides) level. **(D)** Hepatic TG level. Data are expressed as mean ± SD (*n* = 8). *p < 0.05, **p < 0.01, ***p < 0.001 (* represents HFD groups compared with the WT group). ^**#**^ *p* < 0.05, ^**##**^ *p* < 0.01, ^**###**^ *p* < 0.001 (^**#**^ represents ATR treatments compared with the HFD group). WT = control group, and HFD = high-fat diet group.

The livers of HFD feed mice were paler in colors than those of WT mice, but the colors of the livers in the ATR group were redder (Figure 2A, up channel) than those in the HFD mice. On histological analysis, the score of lobular inflammation and hepatocellular ballooning both were 0, as no obvious changes were observed in the H&E staining (Figure 2A, middle channel). Additionally, the ratios of steatosis were all between 5 and 33% in the liver of HFD mice, and the steatosis scores were all 1. Thus, the NAS were the weighted value of steatosis score (the value is 0.375 for all, no significant impact of ATR was observed). The intracellular lipid was analyzed *via* oil red O staining-lipid droplets (LDs; Figure 2A, down channel), and the results showed that LDs in HFD mice liver (area ratio of oil red O is 13%; Figure 2B) were significantly higher than that in WT mice (area ratio of oil red O is 0.3%; Figure 2B); however, the LDs in HFD mice liver were reduced in the presence of ATR, the area ratio of oil red O is 8.25% for low-ATR, and 6.37% for high-ATR (Figure 2B). Furthermore, the serum TG level in HFD mice was significantly reduced by a low concentration of ATR (*p* < 0.05; Figure 2C), and the hepatic TG level was decreased by ATR, both in the low and high concentration of ATR (*p* < 0.05; Figure 2D). Additionally, fibrosis of the liver was analyzed by Masson trichrome staining, the results showed that the hepatic fibrosis of HFD mice was observed in perisinusoidal or periportal space (Supplementary Figure S1B), and about 0.79% fibrosis was measured (Supplementary Figure S1B). ATR treatment decreased hepatic fibrosis, in both low (0.61% fibrosis) and high concentration (0.35% fibrosis) (Supplementary Figure S1B). Altogether, these results implied that ATR can improve the NAFLD steatosis and decrease HFD-induced lipid accumulation in the liver.

### Changes in ANT-AMPK-mTORC1 Signaling Pathway Induced by Atractyloside

**FIGURE 3:**
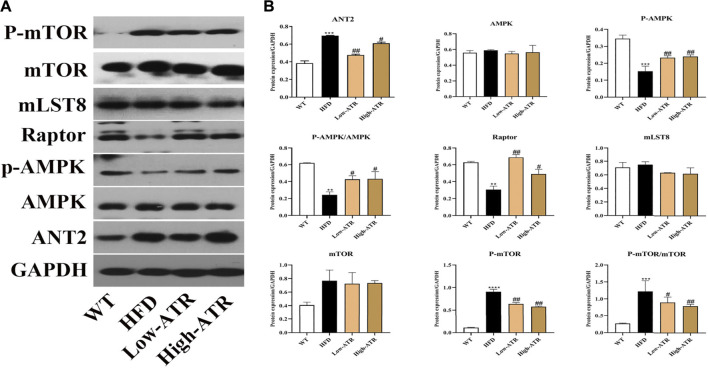
The regulation of ANT-AMPK-mTORC1 signaling pathway by ATR. **(A)** Immunoblot analysis results of ANT2, AMPK, phosphorylated AMPK (p-AMPK), Raptor, mLST8, mTOR, and phosphorylated mTOR (p-mTOR). **(B)** The relative protein expression in reference to GAPDH. Data are expressed as mean ± SD (*n* = 8). *p < 0.05, **p < 0.01, ***p < 0.001 (* represents HFD groups compared with the WT group). ^**#**^ *p* < 0.05, ^**##**^ *p* < 0.01, ^**###**^ *p* < 0.001 (^**#**^ represents ATR treatments compared with the HFD group). WT = control group and HFD = high-fat diet group.

As shown in Figure 3, HFD treatment increased the protein expression of ANT2 (*p* < 0.05) and decreased the p-AMPKα protein expression, the value of p-AMPKα/AMPKα, and the Raptor expression (*p* < 0.05 for all); furthermore, HFD treatment increased the protein expression of p-mTOR and the value of p-mTOR/mTOR (*p* < 0.05 for all). In contrast to the HFD group, ATR treatment decreased the ANT2 protein expression (*p* < 0.05) and increased the p-AMPKα protein expression, the value of p-AMPKα/AMPKα, and the Raptor expression (*p* < 0.05 for all); moreover, ATR decreased the protein expression of p-mTOR and the value of p-mTOR/mTOR (*p* < 0.05 for all). No significant change was observed in the protein expression of mLST8.

### Activation of Autophagy Induced by Atractyloside

**FIGURE 4:**
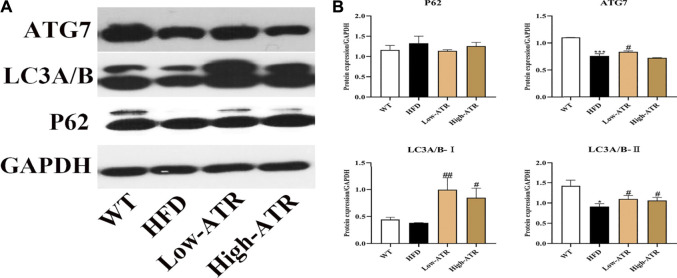
The autophagy induced by ATR in HFD mice. **(A)** Immunoblot analysis results of P62, LC3A/B, and ATG7. **(B)** The relative protein expression in reference to GAPDH. Data are expressed as mean ± SD (*n* = 8). *p < 0.05, **p < 0.01, ***p < 0.001 (* represents HFD groups compared with the WT group). ^**#**^ *p* < 0.05, ^**##**^ *p* < 0.01, ^**###**^ *p* < 0.001 (^**#**^ represents ATR treatments compared with the HFD group). WT = control group and HFD = high-fat diet group.

The protein expressions related to autophagy were determined to analyze the role of autophagy in liver steatosis induced by HFD. As shown in Figure 4, HFD treatment decreased the protein expression of ATG7 and LC3A/B-Ⅱ (*p* < 0.05 for all). In contrast to the HFD group, ATR treatment increased the protein expression of LC3A/B-Ⅱ both in the low and high concentration of ATR (*p* < 0.05), and only a high concentration of ATR increased the ATG7 protein expression (*p* < 0.05). No significant change was observed in the protein expression of P62.

### Colocalization of LC3B Protein With Perilipin 2 Protein

**FIGURE 5:**
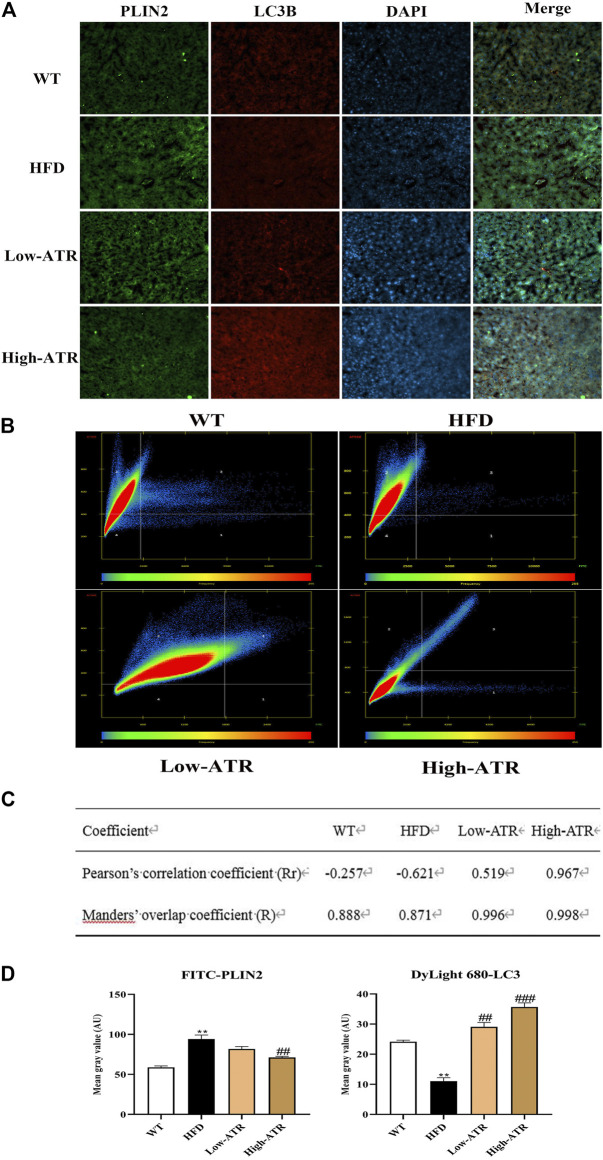
The colocation of LC3B protein with perilipin 2 protein. **(A)** Representative confocal images of colocalization of Cy3-stained LC3B protein with FITC-stained PLIN2 protein. **(B)** The scatter plot obtained from Colocalization modules of ZEN software. Quadrant 1 represents pixels that have high green intensities and low red intensities. Quadrant 2 represents pixels that have high red intensities and low green intensities. Quadrant 3 represents the colocalized pixels with high intensity levels in both red and green. Quadrant 4 represents the background pixels with low intensity levels in both red and green. **(C)** Results of coefficients calculations; value of Rr expressed from −1.0 to +1.0, value from 0.5 to 1.0 indicating colocalization, and value from −1.0 to 0.5 indicating absence of colocalization; value of R expressed from 0 to 1.0, the value indicates an actual overlap of the signals, is considered to represent the true degree of colocalization. **(D)** Optical density analysis of fluorescence intensity. Data are expressed as mean ± SD (*n* = 8). *p < 0.05, **p < 0.01, ***p < 0.001 (* represents HFD groups compared with the WT group). ^**#**^ *p* < 0.05, ^**##**^ *p* < 0.01, ^**###**^ *p* < 0.001 (^**#**^ represents ATR treatments compared with the HFD group). WT = control group, and HFD = high-fat diet group.

Colocalization of LC3B and Perilipin 2 (PLIN2) was performed to determine whether the reduced LDs were associated with autophagy or not. As shown in Figure 5A, the liver slice of HFD mice exhibited a limited amount of colocalization of LC3B and PLIN2 (Figure 5A, row 2), as evidenced by the coefficient of Rr (−0.621) in the HFD group (Figures 5B,C). Meanwhile, the liver co-treated with HFD and ATR showed an increased amount of colocalization of LC3B and PLIN2 (Figure 5A, row 3 and 4), as indicated by the coefficients of Rr (0.519 for low-ATR and 0.967 for high-ATR) in the ATR group (Figures 5B,C). Besides, the optical density analysis showed that ATR treatment reduced the green fluorescence of FITC-stained PLIN2 and increased the red fluorescence of Cy3-stained LC3B (Figure 5D; *p* < 0.05 for all). These data revealed the direct association between LC3B and PLIN2 in ATR-treated HFD mice livers.

## Discussion

ATR, a diterpenoid glycoside known for its specific oxidative phosphorylation inhibitory effect, is found in the fruits of *Xanthium Sibiricum* (Stewart and Steenkamp, 2000). ATR is a high-affinity specific inhibitor of ANT; the depletion or chemical inhibition of ANT2 improves the liver steatosis by reducing hepatic TG level (Cho et al., 2017), implying the potential effect of ATR on NAFLD treatment. In addition, the previous study revealed the effect of ATR on reducing the accumulation of TG level in steatosis of HepG2 cells by activation of autophagy (Zhang et al., 2020). In this study, we further confirmed the therapeutic effect of ATR on NAFLD mice induced by HFD.

High-fat diet feeding for 8 weeks can cause hepatic steatosis and dyslipidemia, which is also confirmed in the present study, as evidenced by the elevated body weight, relative weight (RW) of liver and epididymal fat, and serum AST level in the HFD group (Figure 1). These biochemical parameters are often tested together to illustrate the basic metabolic changes and liver tissue damage induced by HFD (Shi et al., 2019). The low level of serum AST level, RW of liver and epididymal fat, and body weight in ATR-treated mice indicated the relieving effect of ATR on HFD induced basic metabolic changes.

Hepatic lipid accumulation is the main cause for hepatic metabolic disorder, which also may induce lipid peroxidation, oxidative stress, and inflammation in the liver (Rinella, 2015). Hepatic histological analysis showed no lobular inflammation, hepatocellular ballooning, and slight fibrosis, and the steatosis ratios of the livers were all between 5 and 33% (Figure 2B). Thus, this can be defined as hepatocyte steatosis. However, the change of TG level in serum and liver would be a significant parameter to reflect the hepatic steatosis level. The decreased LDs (oil red O staining) and TG level in serum and liver observed in the ATR-treated HFD mice implied that ATR promotes the degradation of accumulated lipid in the liver induced by HFD. This result is consistent with the previous study reporting that ATR can alleviate FFA-induced lipid accumulation in HepG2 cells (Zhang et al., 2020).

With the exchange of ADP and ATP between the cytoplasm and mitochondrial by ANT, the protons will flow back to the mitochondrial matrix (Ruprecht and Kunji, 2020). Thus, excessive ANT activity will cause proton leakage from the intermembrane space, leading to uncoupled respiration (Lee et al., 2014). The present study showed that the ANT2 expression is increased in the liver of the HFD mice group, but ATR treatment decreased the protein expression of ANT2. Combined with the elevated ADP/ATP ratio (result from the *in vitro* cell experiment), these results meet the basic condition for the activation of AMPK. The activation of AMPK regulates lipid metabolism, and its inhibition is associated with steatosis in lipid-overload conditions (Ke et al., 2018). In this study, we found that ATR significantly increased the protein expression of p-AMPKα and increased the ratio of p-AMPKα/AMPKα, suggesting the activation of AMPK induced by ATR. Activated AMPK directly phosphorylates TSC2, enhances its GAP activity, and thereby leads to the inhibition of mTORC1, which is a complex composed of mTOR, Raptor, and mLST8 (also known as GβL) (Wullschleger et al., 2006). ATR exerted a similar effect in the current study, which decreased the protein level of P-mTOR and the value of p-mTOR/mTOR. Interestingly, the protein level of Raptor was increased by ATR, but Raptor is normally considered as a positive role in the nutrient-stimulated mTOR signaling pathway. However, under nutrient-deprived or mitochondrial uncoupling conditions, Raptor also serves as a negative regulator of mTOR kinase activity (Kim et al., 2002). Altogether, these results suggested that ATR inhibits ANT2 expression, promotes the activation of AMPK, increases Raptor expression, and finally decreases the mTOR activity. This would be a potential for autophagy activation.

To analyze the role of autophagy in the current study, western blot was used to detect the expression of autophagy related protein P62, LC3A/B, and ATG7. These proteins are closely connected with the number of autophagosome and act as good symbols of autophagosomes formation (Martinez-Lopez and Singh, 2015). The present results showed that expressions of LC3A/B-Ⅰ, LC3A/B-Ⅱ, and ATG7 were increased in ATR treated HFD mice, which implies the promotion of autophagic activation. The activation of autophagy can directly phagocyte lipid droplets and transport them to lysosomes for degradation. Thus, we further detected the colocalization of LC3B with PLIN2 (the main protein on the surface of LDs); this can confirm the link between ATR-induced autophagy and decrease lipid content. The liver in ATR-treated HFD mice showed increased LC3B and decreased PLIN2, as indicated by the increased red fluorescence and decreased green fluorescence (Figure 5D). Furthermore, the coefficients of Rr (0.519 for low-ATR and 0.967 for high-ATR) in ATR groups suggested a strong colocalization relationship between autophagy and lipid droplets in the liver. Therefore, ATR-medicated autophagy was confirmed to promote the degradation of accumulated lipids in the liver induced by HFD.

In summary, the present study demonstrated that ATR treatment blocks ANT2 expression, promotes the activation of AMPK, then decreases the mTOR activity, and finally promotes autophagy activation, thus accelerating the degradation of accumulated lipids in the liver induced by HFD. This will provide new therapeutic ideas and experimental data for clinical prevention and treatment of NAFLD.

## Data Availability

The raw data supporting the conclusions of this article will be made available by the authors, without undue reservation.
